# Characterization and machine learning analysis of hybrid alumina-copper oxide nanoparticles in therminol 55 for medium temperature heat transfer fluid

**DOI:** 10.1038/s41598-025-92461-3

**Published:** 2025-03-11

**Authors:** G. Kadirgama, D. Ramasamy, K. Kadirgama, L. Samylingam, Navid Aslfattahi, Mohammad Reza Chalak Qazani, Chee Kuang Kok, Talal Yusaf, Michal Schmirler

**Affiliations:** 1 Faculty of Mechanical and Automotive Engineering Technology, Universiti Malaysia Pahang, Pekan, 26600 Pahang Malaysia; 2 Center of Excellence for Advanced Research in Fluid Flow, Universiti Malaysia Pahang, Pekan, 26600 Pahang Malaysia; 3 Centre for Sustainable Materials & Surface Metamorphosis (CSMSM), Chennai Institute of Technology, Chennai, India; 4 Centre for Advanced Mechanical and Green Technology, Faculty of Engineering and Technology, Multimedia University, Jalan Ayer Keroh Lama, Bukit Beruang, Melaka, 75450 Malaysia; 5 Institute of Fluid Dynamics and Thermodynamics, Faculty of Mechanical Engineering, Czech Technical University in Prague, Technická 4, Prague, 166 07 Czech Republic; 6 Faculty of Computing and Information Technology, Sohar University, Sohar, Oman; 7 School of Engineering and Technology, Central Queensland University, Brisbane, QLD 4008 Australia

**Keywords:** Engineering, Materials science

## Abstract

Efficient heat dissipation is crucial for various industrial and technological applications, ensuring system reliability and performance. Advanced thermal management systems rely on materials with superior thermal conductivity and stability for effective heat transfer. This study investigates the thermal conductivity, viscosity, and stability of hybrid Al_2_O_3_-CuO nanoparticles dispersed in Therminol 55, a medium-temperature heat transfer fluid. The nanofluid formulations were prepared with CuO-Al_2_O_3_ mass ratios of 10:90, 20:80, and 30:70 and tested at nanoparticle concentrations ranging from 0.1 wt% to 1.0 wt%. Experimental results indicate that the hybrid nanofluids exhibit enhanced thermal conductivity, with a maximum improvement of 32.82% at 1.0 wt% concentration, compared to the base fluid. However, viscosity increases with nanoparticle loading, requiring careful optimization for practical applications. To further analyze and predict thermal conductivity, a Type-2 Fuzzy Neural Network (T2FNN) was employed, demonstrating a correlation coefficient of 96.892%, ensuring high predictive accuracy. The integration of machine learning enables efficient modeling of complex thermal behavior, reducing experimental costs and facilitating optimization. These findings provide insights into the potential application of hybrid nanofluids in solar thermal systems, heat exchangers, and industrial cooling applications.

## Introduction

Efficient heat dissipation is critically important for various industrial and technical purposes. It is critical in ensuring the reliability, efficacy, and longevity of systems ranging from microelectronics to huge-scale industrial machinery^1^. The value of advanced thermal management systems is highly significant since they directly assess the performance and energy use of equipment and procedures in the current competitive technological context. The main issue revolves around the generation and buildup of thermal energy, which occurs during electrical and mechanical processes^2^. Without regulation, the accumulation of heat by an incompetent heating system can result in overheating and a decrease in efficiency or even complete system failure in severe cases. Within industries, machines and operations produce a significant quantity of heat that must be controlled as an integral part of the system and for safety purposes^3^.

Aluminium oxide (Al_2_O_3_), is a chemical compound with unique thermal properties that render it a naturally crucial material for thermal management needs in many sectors^4^. The most significant component of modern thermal management solutions is the inherent characteristics of aluminium oxide, such as its thermal resistance, thermal conductivity, and resistance to thermal shock^5^. Pure alumina does not chemically react with other elements for various materials, but it withstands up to 2072 °C for others. The reason behind the high melting point of alumina is the covalent bond formation between the aluminium and the oxygen atoms^6^. Aluminium oxide is highly conductive to heat, a better attribute than many other ceramics. Thermal conductivity is the ability of a material to carry heat quantitatively^7^. Aluminium oxide’s thermal conductivity varies from 20 to 30 W/(m·K), depending on its purity and grain size^8^. The ability of alumina to carry thermal energy makes it one of the major deciding factors in choosing pan materials, such as heat sinks that need to conduct heat quickly to manage the reception of other tools like electronic devices.

Copper oxide (CuO), with its outstanding thermal properties, has become the focus of great interest in the thermal management sector, particularly when applies in nanoparticle form^9^. Nanoparticles possess several unique features, such as enhanced thermal conductivity and specific heat capacity, that make them ideal for thermal management^10^. In addition to the nanoparticle’s innate thermal characteristics, the high surface area to volume ratio contributes to and promotes greater efficiency^11^. CuO nanoparticles have high thermal conductivity, making them suitable for dispersing in a base fluid. Consequently, the thermal performance of a fluid increases by promoting heat transfer and making it more efficient. According to the outcomes, nanoparticles’ ability to do a mostly singular network allows excellent heat transfer to be acquired^12^. Adding CuO nanoparticles into water and ethylene glycol improves thermal conductivity through uniform dispersion. As a result, heat is efficiently transferred through custom services to additional services, including thermal interface materials and coolants. The increased thermal conduction efficiency is likely due to the high conductive nature of nanoparticles and the motion of the base fluid around the dispersed nanoparticles^13^.

The application of alumina and copper oxide in hybrid material formation for thermal management has lately become quite a popular topic for applications requiring high thermal conductivity and heat dissipation^14–16^. This is primarily due to the high-temperature resistance of alumina and heat conductibility for copper oxide^17^. The observed synergy in the case of alumina-copper oxide hybrids is due to the high thermal conductivity of copper oxide and the good thermal stability of alumina^17^. When these two features are united in one material, it will possess a higher thermal conductivity than both from the comparison. The higher efficiency is due to the ease with which the added copper oxide particles incorporated in the alumina frame create conductive paths to quickly dissipate the heat^18^. Additionally, embedding copper oxide into alumina helps reduce thermal resistance at the interface of composite materials and thus increases the overall efficiency of heat transmission^17^. Proper thermal management of active components is crucial to their performance and reliability at all times, and it becomes even more critical in such applications as electronic packaging. Alumina-copper oxide hybrids have been explored only little in terms of the efficiency in thermal quantification. For example, Turco et al.^19^ worked with alumina and copper oxide composites to determine the material’s thermal conductivity level. In their research, adding copper oxide greatly increased thermal conductivity efficiency. Research has shown that the best balance of copper oxide dispersed in the alumina matrix is more effective than pure alumina and copper oxide itself.

Therminol 55’s has a high boiling point at standard atmospheric pressure, meaning it may be used at higher temperatures without the need for unreasonable pressure levels^20^. To improve heat efficiency, the fluid comprises certain heat capacity and thermal conductivity at specific rates. Naturally, the substance is best used for a broad range of operating temperatures, generally from − 15 °C to 315 °C (5 °F to 600 °F)^21^. Nevertheless, Therminol 55 has excellent characteristics at low temperatures, making it superb for pumping and system start-up^22^. Solar thermal power plants employ a heat transfer fluid (HTF) to transport thermal energy from solar collectors to power generation equipment. The system’s high-temperature stability reduces the likelihood of deterioration and ensures consistent performance, which is crucial for the efficiency and longevity of these systems^21^.

Research has primarily focused on investigating the compatibility of Therminol 55 with different types of nanoparticles, including metallic (such as copper and silver), oxide (such as alumina and silica), and carbon-based (such as graphene and carbon nanotubes)^22–26^. Nanoparticles greatly increase the base fluid’s ability to transfer heat due to the significant increase in thermal conductivity. The study by Yu et al.^27^ demonstrated that the thermal conductivity values for Therminol 55 with the addition of copper oxide nanoparticles may reach up to 20%. Thus, the efficiency of the thermal systems is increased. Therminol 55 is widely used in practice due to its high temperature. Remarkably, the fluid exhibits excellent thermal stability, which implies that it can perform acceptably at temperatures close to the maximum temperatures before marking material degeneration^28^. Consequently, it can be used for long periods at high temperatures. Additionally, using the fluid at high temperatures decreases the thermal system’s frequency of maintenance since it is not prone to cradle, solidify, or boil.

Previous research has primarily focused on single nanoparticle suspensions, such as Al_2_O_3_, CuO, TiO_2_, and carbon-based materials, evaluating their impact on thermal conductivity, viscosity, and stability^29,30^. However, limited studies have explored the thermo-optical characterization of hybrid nanofluids based on Therminol 55, particularly those incorporating metal oxide hybrids like Al_2_O_3_-CuO. Hybrid nanofluids have shown synergistic effects, where the combination of different nanoparticles leads to enhanced thermal conductivity and stability compared to single-component nanofluids^31^. Despite these advantages, there remains a lack of comprehensive studies analyzing the combined influence of concentration, hybrid mass ratios, and temperature on the thermophysical properties of Therminol 55 nanofluids^32^. Furthermore, most previous studies rely solely on experimental data, whereas this study integrates machine learning for predictive modeling, offering a more efficient approach to optimizing thermal performance^33^. By addressing these gaps, this research provides a detailed thermo-optical characterization of Al_2_O_3_-CuO hybrid nanofluids in Therminol 55 while introducing a Type-2 Fuzzy Neural Network (T2FNN) model to predict thermal conductivity, reducing experimental dependency and improving optimization capabilities.

The objective was to explore a novel method to increase heat transfer efficiency for medium-temperature applications. Furthermore, this research was designed to promote thermal management technologies through a comprehensive analysis of the impact of enhanced nanoparticles on fluidic thermal conductivity and viscosity. In addition to the characterization and performance analysis of developed nanofluids, this research introduces a novel approach to augmenting heat transfer efficiency in medium-temperature applications. Leveraging machine learning techniques, specifically a Type-2 Fuzzy Neural Network (T2FNN), thermal conductivity prediction becomes more accurate and insightful. By incorporating temperature, concentration, and weight ratios as input parameters, the T2FNN model offers a sophisticated means of understanding the complex interplay of factors influencing thermal conductivity. This integration of machine learning enhances the depth of analysis and opens avenues for optimizing heat transfer processes beyond traditional methodologies. Through this innovative approach, this study aims to contribute to advancing thermal management technologies, paving the way for more efficient and reliable cooling systems tailored to the demands of modern technological applications.

## Methodology

### Preparation

The first step is to prepare the materials. High-purity Aluminium oxide nanoparticles and copper oxide are bought from US Research Nanomaterials, Inc. (Houston, TX, USA). The purchased items are available for use in 20 to 50 nm dimensions. This range is set to control the dispersion stability and the possible increase in the thermal conductivity of the solution. Merck, India, has provided the Therminol 55. Synthesis of nanofluid, involves precisely measuring the weight of alumina and copper oxide nanoparticles to achieve a desired concentration in the fluid, such as 0.1 weight per cent. Then, the Therminol 55 was measured according to the weight% of nanoparticles that needed to be added. Next, the nanoparticles are introduced into the Therminol 55 while stirred continuously at 400 rpm and 50 ℃ on a hot plate magnetic stirrer. Next, to ensure a consistent distribution, the mixture undergoes ultrasonication using an ultrasonic probe at 70% power for an hour with 7s on and 3s off^34^.

**Fig. 1:**
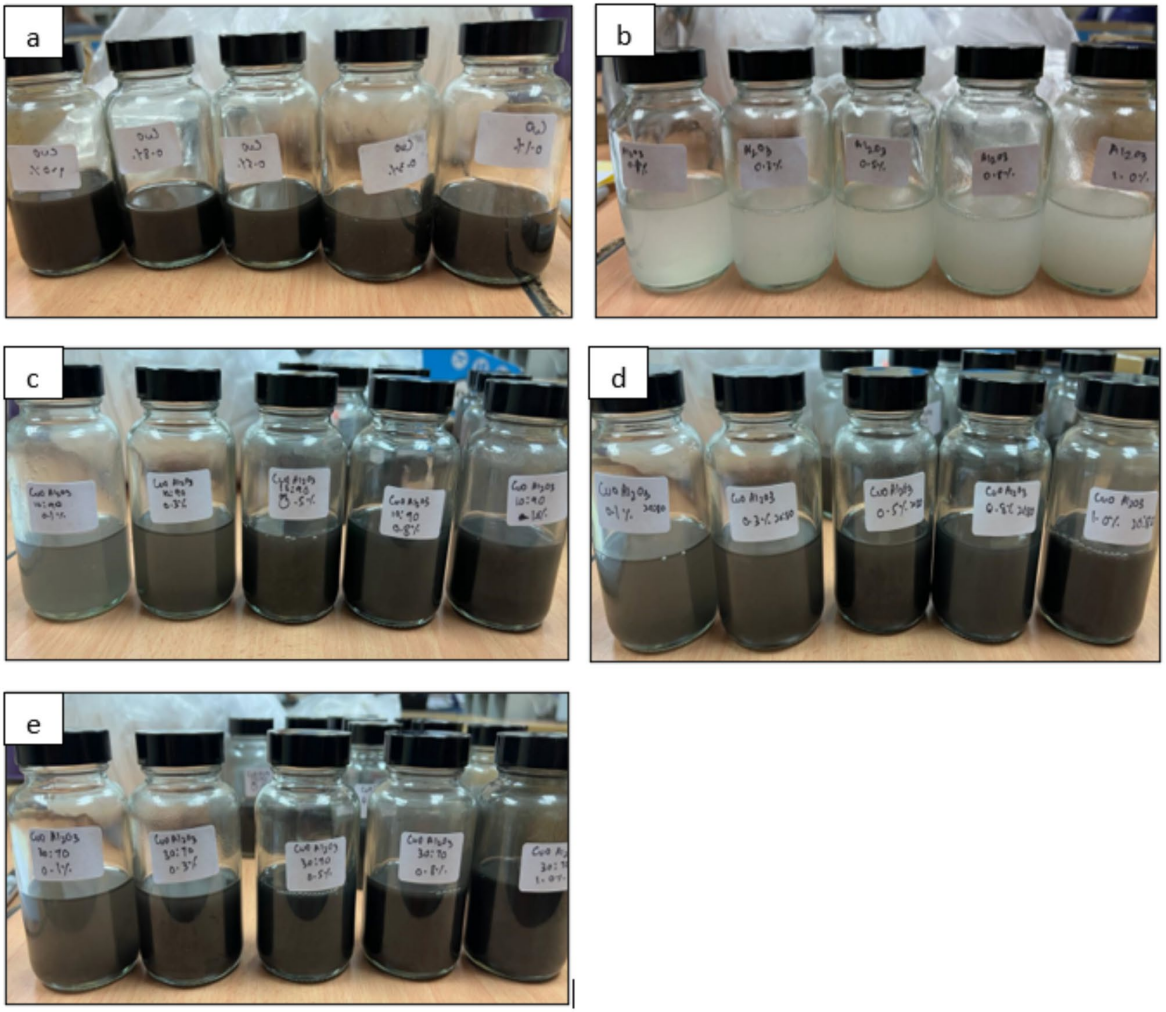
(**a**) CuO-Therminol 55 nanofluid with concentration 0.1wt%, 0.3wt%, 0.5wt%, 0.8wt% and 1.0wt%. (**b**) Al_2_O_3_-Therminol 55 nanofluid with concentration 0.1wt%, 0.3wt%, 0.5wt%, 0.8wt% and 1.0wt%. (**c**) CuO- Al_2_O_3_ (10:90) hybrid Therminol 55 nanofluid with concentration 0.1wt%, 0.3wt%, 0.5wt%, 0.8wt% and 1.0wt%. (**d**) CuO- Al_2_O_3_ (20:80) hybrid Therminol 55 nanofluid with concentration 0.1wt%, 0.3wt%, 0.5wt%, 0.8wt% and 1.0wt%. (**e**) CuO- Al_2_O_3_ hybrid (30:70) Therminol 55 nanofluid with concentration 0.1wt%, 0.3wt%, 0.5wt%, 0.8wt% and 1.0wt%.

The hybrid nanofluids were prepared by dispersing CuO and Al_2_O_3_ nanoparticles in Therminol 55 at weight concentrations of 0.1, 0.3, 0.5, 0.8, and 1.0 wt%. The hybrid compositions were formulated with CuO-Al_2_O_3_ weight ratios of 10:90, 20:80, and 30:70, ensuring a systematic variation in nanoparticle content to evaluate their influence on thermal properties. To verify the accuracy of the prepared concentrations and ensure proper dispersion, UV-Vis spectroscopy was conducted to assess nanoparticle stability and uniformity in the base fluid. These characterization techniques confirm that the stated weight ratios and concentrations were accurately prepared and consistently maintained throughout the study. Furthermore, previous studies utilizing similar weight ratios for hybrid metal oxide nanofluids have demonstrated effective stability and improved heat transfer performance, supporting the reliability of the selected formulations^35–37^. Figure 1 shows all the samples that were synthesized for the experimental work.

### Characterization

#### Morphology and optical properties

The surface texture of the formed Al_2_O_3_-CuO nanoparticles was inspected with a scanning electron microscope (SEM). The picture was taken in high vacuum conditions, and a landing voltage of 10 kV was applied. The high magnification allows observing the nano-scale features of the nanoparticles. The microscope resolution lies in the micrometer range. Fourier transforms infrared (FTIR) spectroscopy was used to identify the chemical conformations of the formulated samples. The device was operated at a 0.2 scan speed for each spectrum while the resolution was set at 4 cm^− 1^ resolution. The spectral wavelength ranged from 500 to 4000 cm^− 1^. The optical absorbance and transmittance were obtained using a UV–vis spectrometer. The wavelength range of 200 to 800 nm was chosen because it encompasses more than 80% of the total solar energy released by the sun.

### Thermophysical properties

The thermal conductivity (TC) measurement was accomplished using the transient hot-wire method, employing a Tempos thermal property analyzer. The apparatus can assess TC values with an accuracy of 90% or higher. The sample was maintained at a constant temperature during the measurement by placing it in a water bath. The sensed TC was converted into a digital signal and displayed on the monitor by dipping a single heated needle inside the sample, which served as a KS-3 sensor. As the sample temperature reached the anticipated value, the samples were left to equilibrate for at least 30 min before taking the measurement. Five readings were taken to check the repeatability at each point, and mean values were recorded to preserve measurement accuracy. The viscosity and the shear property (shear stress and shear rate) were measured with a rheometer (MCR 92, Anton Paar, Austria). The measurement was assessed at 100 rpm with an accuracy of ± 1.0% in the 30 to 80 ℃ temperature range. To ensure the reliability of the measured thermal conductivity values, an uncertainty analysis was conducted based on the propagation of errors in experimental measurements. The uncertainty was calculated using the standard error propagation formula in Eq. (1)^38^:

1 $$\:{U}_{y}=\sqrt{{\sum\:}_{i=1}^{n}{\left(\frac{\partial\:y}{{\partial\:x}_{i}}\:{U}_{{x}_{i}}\right)}^{2}}$$

where $\:{U}_{y}\:$is the total uncertainty in the measured thermal conductivity, $\:{x}_{i}\:$represents each experimental variable, and $\:{U}_{{x}_{i}}$​​ is the uncertainty associated with each variable. The uncertainty in thermal conductivity measurements was evaluated using the standard propagation of uncertainty formula, as outlined in the Guide to the Expression of Uncertainty in Measurement^38^.

### Machine learning

In the following Subsection, the machine learning method is employed to predict the thermal conductivity based on temperature, concentration, and percentage of Cu. It consists of two parts: data preparation and type-2 fuzzy neural network.

#### Data preparation

A meticulous data preprocessing stage was undertaken to ensure our model’s accuracy in predicting thermal conductivity. This involved several crucial steps:

- *Outlier removal*: Data points falling outside predetermined ranges were identified and eliminated initially. If left unaddressed, these outliers could skew the model’s predictions.
- *Normalization*: Following outlier removal, the dataset was normalized to standardize the range of values. This normalization process aimed to enhance the model’s performance by bringing all variables to a comparable scale, thus mitigating complexities within the system. Specifically, each data point was transformed to fit within the range [0,1] using the formula in Eq. (2):
  2 $${x_{Nor}}=\frac{{x - {x_{\hbox{min} }}}}{{{x_{\hbox{max} }} - {x_{\hbox{min} }}}}$$
  where ${x_{\hbox{min} }}$ and ${x_{\hbox{max} }}$ denote the minimum and maximum values of the dataset, respectively.
- *Dataset partitioning*: The preprocessed dataset was divided into training and testing sets. 80% of the dataset was allocated for training to facilitate robust model training and evaluation. In comparison, the remaining 20% was reserved for testing the model’s performance.

#### Type-2 fuzzy neural network

The predictive model was constructed using a Type-2 Fuzzy Neural Network (T2FNN), a fusion of neural network and type-2 fuzzy inference system (FIS) methodologies. This hybrid approach offers several advantages in modeling complex systems:

- *Model architecture*: The T2FNN architecture consists of two main components: the antecedents and the consequents. These components are interconnected to form a comprehensive network that captures intricate relationships within the data.
- *Rule generation*: The T2FNN operates based on multiple type-2 FIS IF-THEN rules, which govern the mapping between input and output variables. These rules are derived from expert knowledge and data-driven insights, ensuring the model’s robustness and adaptability.
- *Training procedure*: Parameters within the T2FNN were optimized through a training process utilizing the dataset under study. This involved iteratively adjusting the network’s weights and biases to minimize prediction errors and improve overall performance.
- *Model visualization*: The functionality of the T2FNN model is visually represented in a schematic depiction, illustrating the flow of information through the network’s layers and components.

#### Model validation

To assess the reliability and accuracy of the developed model, five key validation parameters were employed:

- *Correlation coefficient (CC)*: Measures the strength and direction of the linear relationship between predicted and actual values as in Eq. (3).
  3 $$CC=\frac{{\sum\limits_{{i=1}}^{n} {\left( {\left( {{x_i} - \bar {x}} \right)\left( {{T_i} - \bar {T}} \right)} \right)} }}{{\sqrt {\sum\limits_{{i=1}}^{n} {\left( {{{\left( {{x_i} - \bar {x}} \right)}^2}{{\left( {{T_i} - \bar {T}} \right)}^2}} \right)} } }}$$
- *Mean Square Error (MSE)*: Quantifies the average squared difference between predicted and actual values, measuring prediction accuracy as in Eq. (4).
  4 $$MSE=\frac{1}{n}{\sum\limits_{{i=1}}^{n} {\left( {{T_i} - {{\hat {T}}_i}} \right)} ^2}$$
- *Root Mean Square Error (RMSE)*: This represents the square root of the MSE, offering a more interpretable measure of prediction error as in Eq. (5).
  5 $$RMSE=\sqrt {\frac{{{{\sum\limits_{{i=1}}^{n} {\left( {{T_i} - {{\hat {T}}_i}} \right)} }^2}}}{n}}$$
  where *n*, ${x_i}$, $\bar {x}$ and $\bar {T}$ are the number of samples, the ith input, the mean of inputs, and the mean of outputs.

These validation parameters were calculated using standard formulas and statistical techniques, enabling rigorous assessment of the model’s performance against empirical data.

In summary, the proposed methodology encompasses rigorous data preprocessing, the construction of a hybrid T2FNN model, and comprehensive validation procedures. This approach ensures the development of a robust and reliable predictive model for thermal conductivity estimation.

## Results and discussion

### SEM analysis

**Fig. 2:**
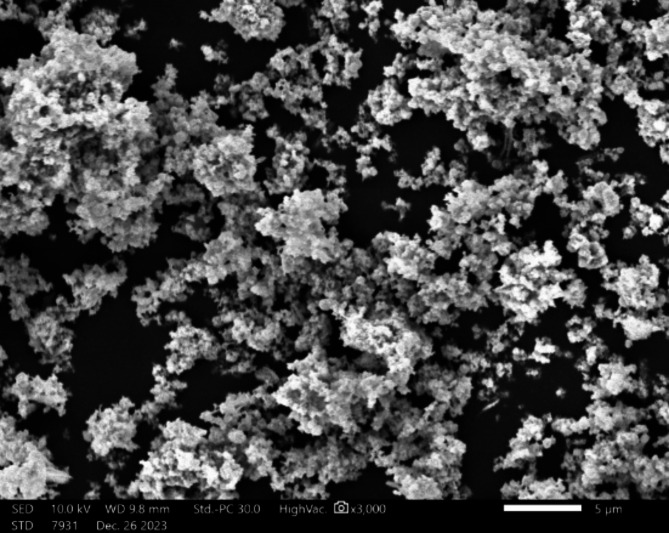
SEM picture of Copper Oxide.

**Fig. 3:**
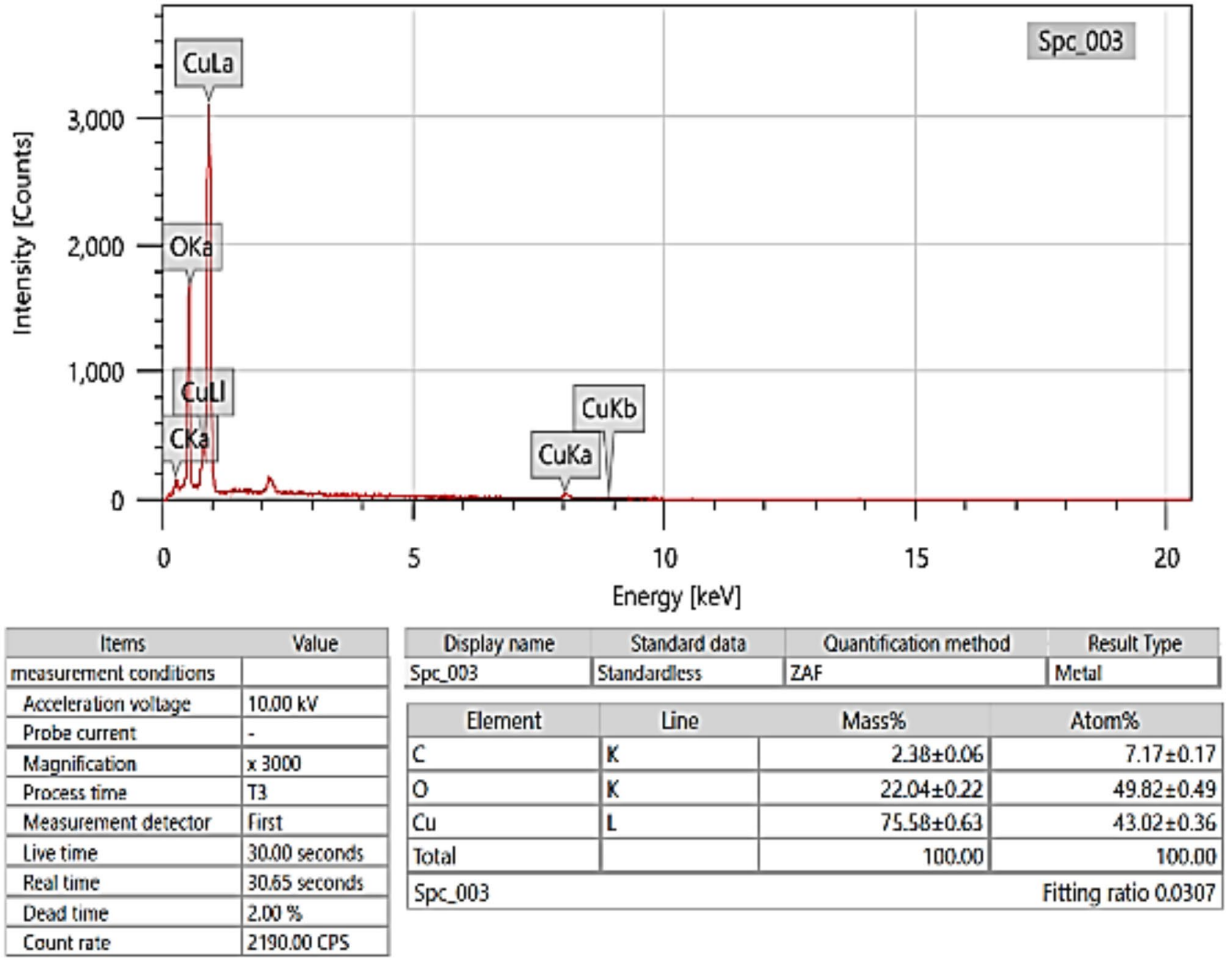
EDX for Copper Oxide.

In Fig. 2, the scanning electron microscope took a picture of copper oxide at 3000x magnification. The image shows high-quality details. The morphologies of the copper oxide in the SEM picture depict agglomerated particle groups. The presence of this particulate consistency is characteristic of numerous synthesized nanoparticles, and similar formations are frequently observed in materials that have undergone procedures such as calcination or precipitation^39^. Figure 3 shows the EDX analysis, which reveals that the sample consists mostly of copper and oxygen, consistent with the composition of copper oxide. Additionally, a small quantity of carbon is detected, which is likely due to external sources such as the carbon tape used^40^. Based on the mass and atomic percentages, it may be inferred that the sample is predominantly made up of copper oxide.

**Fig. 4:**
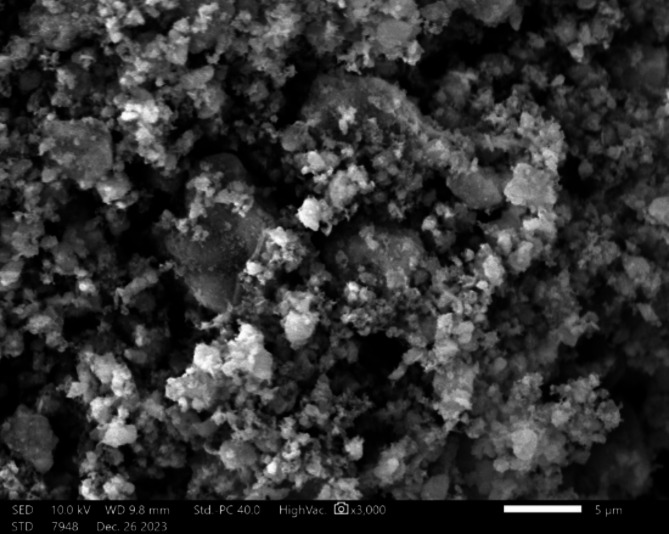
SEM for Aluminium Oxide.

**Fig. 5:**
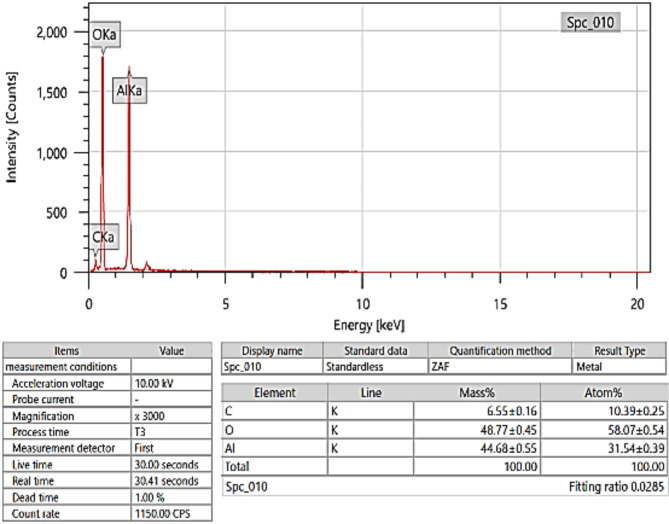
EDX for Aluminium Oxide.

Figure 4 shows an SEM picture of the Aluminium Oxide. The morphology observed in the SEM picture suggests the presence of a conglomeration of particles of diverse sizes and shapes, indicating a polycrystalline nature. This shape is characteristic of aluminium oxide powders, which are extensively utilized in many applications because of their chemical and physical qualities. Based on Fig. 5, the EDX analytical results in the paper, the sample consists of carbon (C), oxygen (O), and aluminium (Al). The mass percentages for carbon, oxygen, and aluminium are roughly 6.55%, 48.77%, and 44.68%, respectively, totaling 100%. When examining the atomic percentages, the sample consists of 10.39% carbon, 58.07% oxygen, and 31.54% aluminium. The carbon detected in the sample may have originated from the sample preparation procedure^40^. Aluminium oxide is a ceramic substance that is recognized for its exceptional hardness, thermal stability, and ability to insulate electricity^41^. It is extensively utilized in technical applications, including as abrasives, refractories, the production of cutting tools, and as a dielectric material in electronics^42^.

**Fig. 6:**
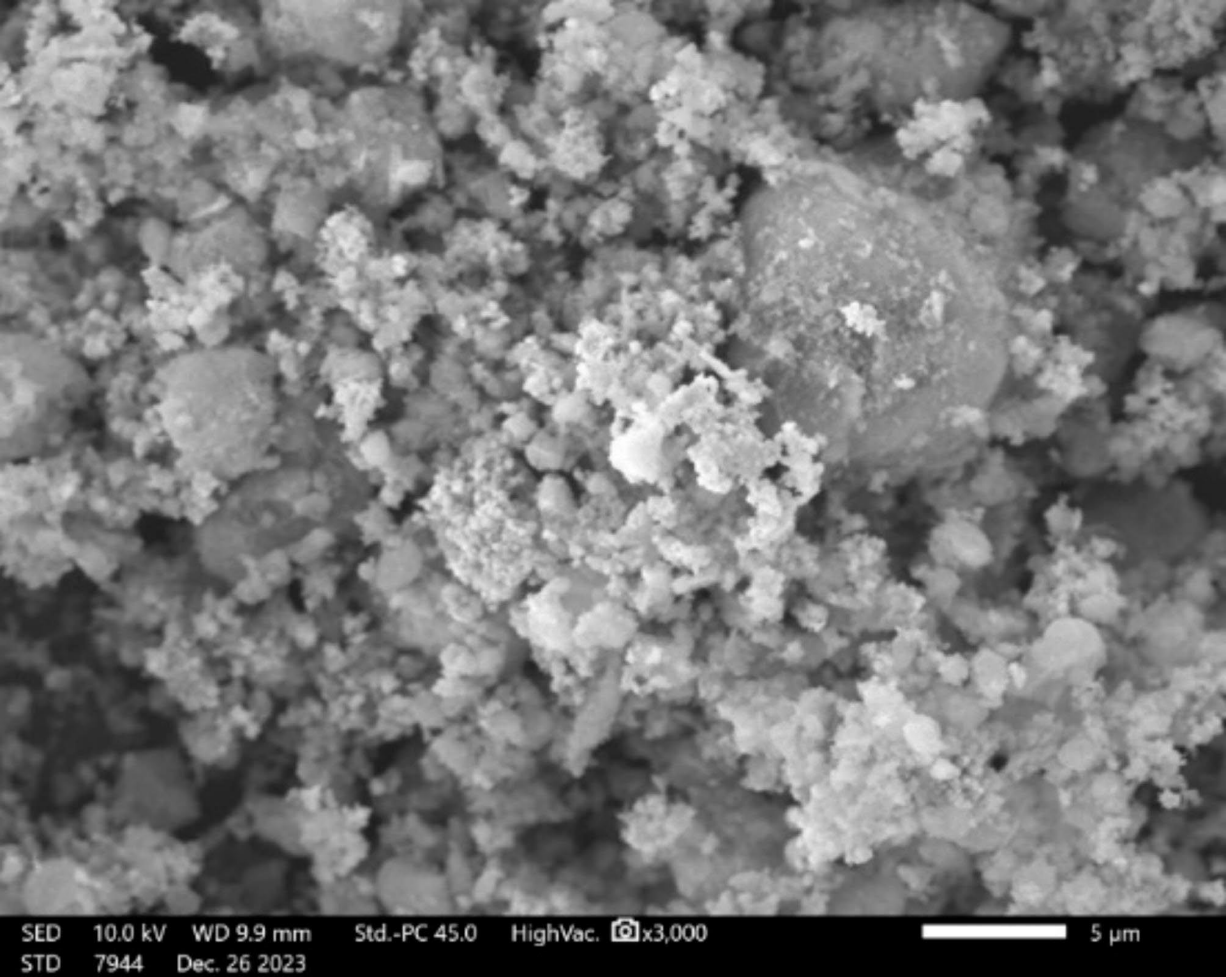
SEM for CuO- Al_2_O_3_ hybrid nanoparticles.

**Fig. 7:**
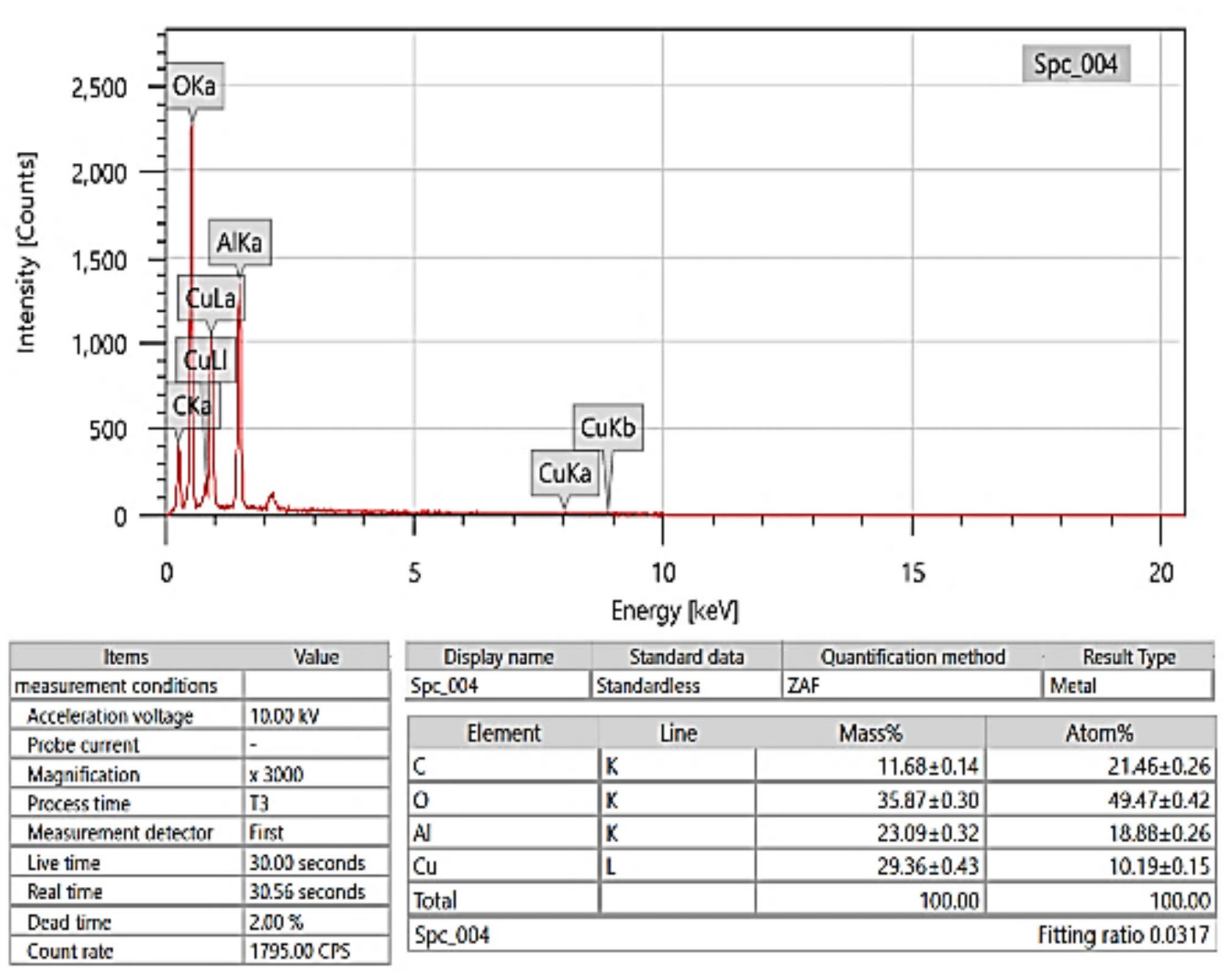
EDX for CuO- Al_2_O_3_ hybrid.

Figure 6 shows the morphology of the hybrid material composed of copper oxide and aluminium oxide. According to Fig. 7, EDX analysis, the material consists of carbon (C), oxygen (O), aluminium (Al), and copper (Cu), with no other elements detected in significant amounts. The mass percentage composition is as follows: 11.68% carbon, 35.87% oxygen, 23.09% aluminium, and 29.36% copper, summing to 100%. The atom percentage composition differs due to the differing atomic weights of the elements, with 21.46% carbon, 49.47% oxygen, 18.88% aluminium, and 10.19% copper. These percentages are standard for hybrid materials where aluminium oxide is a support matrix for copper oxide^43^. The copper oxide particles are likely the active phase, given their role in various catalytic and electronic applications, while the aluminium oxide provides structural stability and surface area^44^. The carbon present could be a residue from the sample preparation method for SEM analysis, which uses carbon tape^40^.

The morphology of nanoparticles significantly influences the thermal conductivity of nanofluids, affecting their heat transfer efficiency, dispersion stability, and interaction with the base fluid^45^. The shape of nanoparticles plays a crucial role, as higher aspect ratio structures provide larger surface contact areas, enhancing phonon and electron transport^46^. In contrast, spherical nanoparticles, such as CuO and Al_2_O_3_, exhibit more isotropic heat transfer but may lead to increased viscosity at higher concentrations. Additionally, nanoparticle size affects heat transfer performance, where smaller nanoparticles exhibit higher Brownian motion and micro-convection effects, improving thermal conductivity^13^. However, excessively small particles may introduce higher interfacial resistance, reducing overall enhancement.

In hybrid nanofluids, the combined morphology of multiple nanoparticles can lead to synergistic effects, improving both thermal conductivity and dispersion stability^47^. In this study, CuO-Al_2_O_3_ hybrid nanoparticles were selected due to their complementary properties, where CuO provides superior thermal conductivity, while Al_2_O_3_ enhances stability and prevents excessive viscosity buildup. This balance ensures an effective heat transfer medium while mitigating issues related to aggregation and sedimentation. The results confirm that the selection of nanoparticle morphology and hybrid composition plays a crucial role in optimizing the thermal performance of nanofluids for industrial applications.

### FTIR analysis

**Fig. 8:**
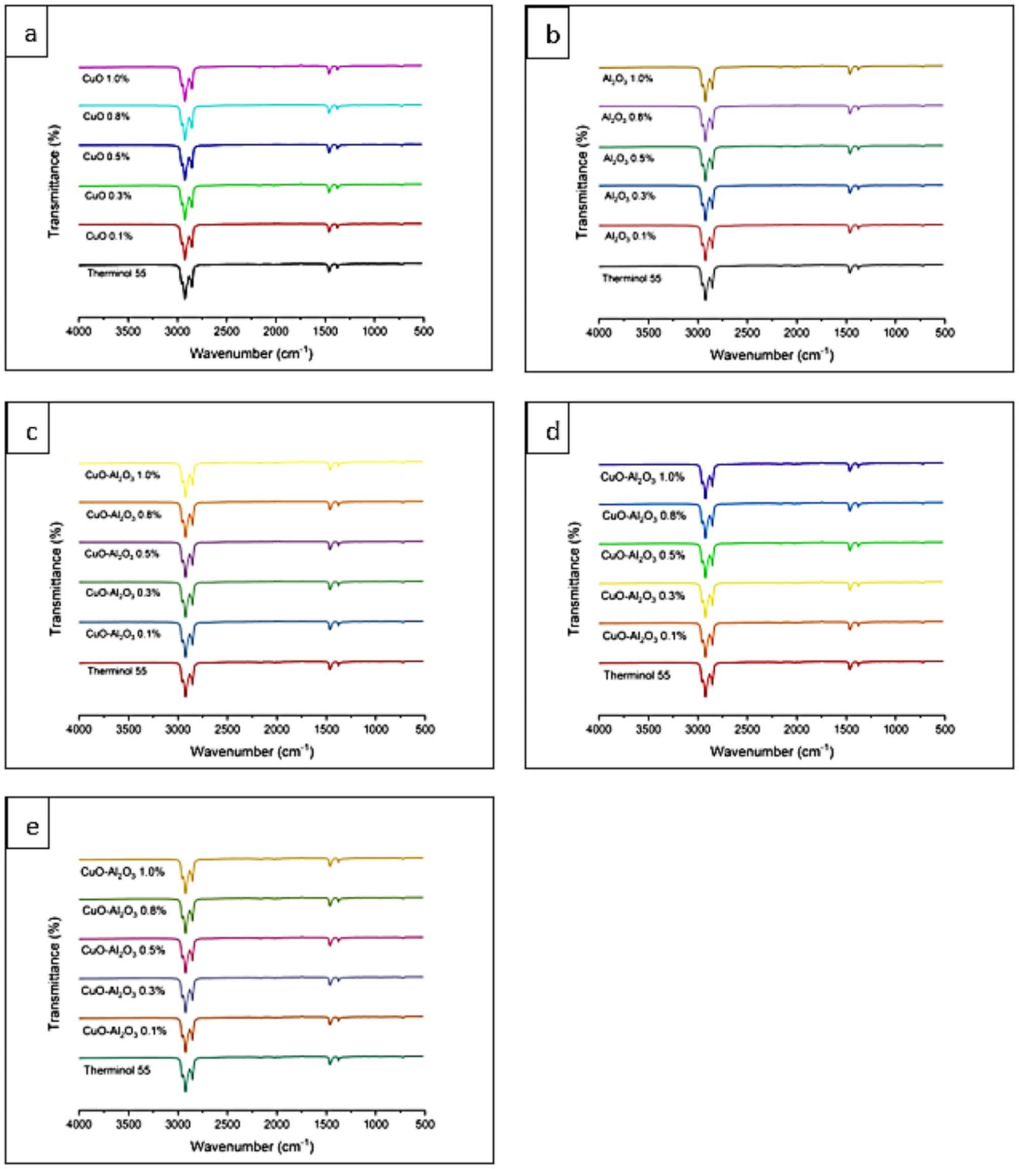
(**a**) FTIR of CuO-Therminol 55 nanofluid with concentration 0.1wt%, 0.3wt%, 0.5wt%, 0.8wt% and 1.0wt%. (**b**) FTIR of Al_2_O_3_-Therminol 55 nanofluid with concentration 0.1wt%, 0.3wt%, 0.5wt%, 0.8wt% and 1.0wt%. (**c**) FTIR of CuO- Al_2_O_3_ (10:90) hybrid Therminol 55 nanofluid with concentration 0.1wt%, 0.3wt%, 0.5wt%, 0.8wt% and 1.0wt%. (**d**) FTIR of CuO- Al_2_O_3_ (20:80) hybrid Therminol 55 nanofluid with concentration 0.1wt%, 0.3wt%, 0.5wt%, 0.8wt% and 1.0wt%. (**e**) FTIR of CuO- Al_2_O_3_ hybrid (30:70) Therminol 55 nanofluid with concentration 0.1wt%, 0.3wt%, 0.5wt%, 0.8wt% and 1.0wt%.

Figure 8 shows the FTIR results for CuO, Al_2_O_3_, and hybrid CuO- Al_2_O_3_ therminol 55 nanofluids. The spectral analysis revealed several notable peaks corresponding to different functional groups. One such vibrational band, observed at 1490 cm^− 1^, indicates C-C bonds. These bonds form the backbone of the carbon skeleton, a fundamental component of many organic molecules, signifying the presence of complex carbon-based structures within the nanofluid^48^. Another significant peak was detected at 2950 cm^− 1^, associated with the O-H stretching vibrations. This characteristic vibration is typical of alcoholic and phenolic groups, suggesting the presence of these functional groups in the nanofluid^49^. These groups are often involved in hydrogen bonding and play a crucial role in a substance’s physical and chemical properties.

A peak observed at 1396 cm-1 is also assigned to C-O-C stretching vibrations. This particular stretching motion is characteristic of ether groups, indicating their presence within the nanofluid’s molecular structure^50^. Ethers are notable for their ether linkage - an oxygen atom connected to two alkyl or aryl groups - which can significantly influence the solubility and stability of the compound^51^. Overall, the FTIR spectroscopy analysis provided a detailed insight into the molecular composition of the copper oxide, aluminium oxide, and copper oxide-aluminium oxide Therminol 55 nanofluid.

The absence of new peaks suggests that the nanoparticles and the base fluid have not undergone any chemical reactions to form new compounds^52^. Chemical stability implies that the nanoparticles remain evenly dispersed within the base fluid without reacting with it or with each other^53^. This stable dispersion is essential for maintaining the nanofluid’s enhanced thermal or physical properties over time.

### UV-Vis analysis

**Fig. 9:**
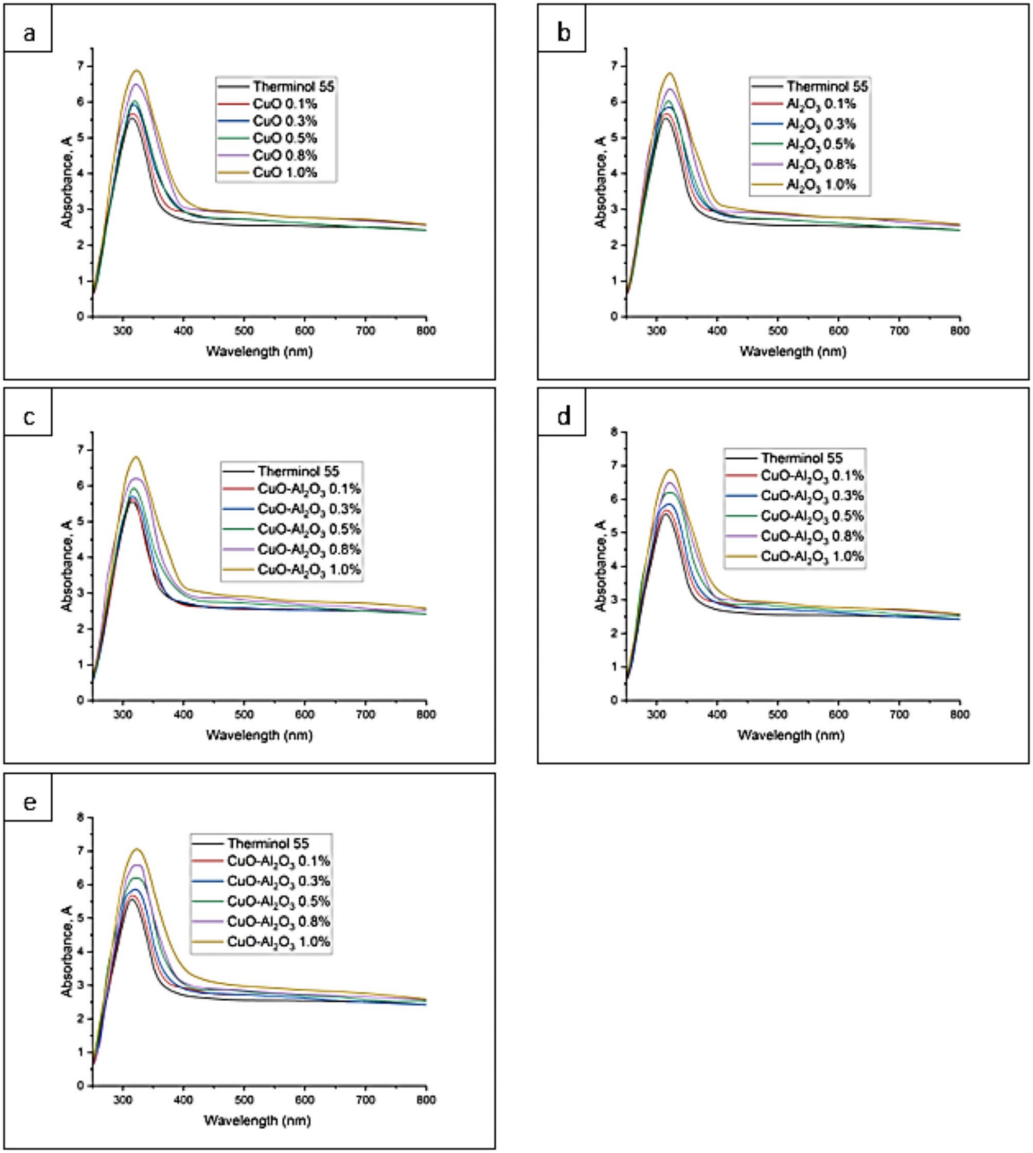
(**a**) UV-Vis of CuO-Therminol 55 nanofluid with concentration 0.1wt%, 0.3wt%, 0.5wt%, 0.8wt%, and 1.0wt%. (**b**) UV-Vis of Al_2_O_3_-Therminol 55 nanofluid with concentration 0.1wt%, 0.3wt%, 0.5wt%, 0.8wt% and 1.0wt%. (**c**) UV-Vis of CuO- Al_2_O_3_ (10:90) hybrid Therminol 55 nanofluid with concentration 0.1wt%, 0.3wt%, 0.5wt%, 0.8wt% and 1.0wt%. (**d**) UV-Vis of CuO- Al_2_O_3_ (20:80) hybrid Therminol 55 nanofluid with concentration 0.1wt%, 0.3wt%, 0.5wt%, 0.8wt% and 1.0wt%. (**e**) UV-Vis of CuO- Al_2_O_3_ hybrid (30:70) Therminol 55 nanofluid with concentration 0.1wt%, 0.3wt%, 0.5wt%, 0.8wt% and 1.0wt%.

Figure 9 shows the UV-Vis results for CuO, Al_2_O_3_, and CuO- Al_2_O_3_ therminol 55 nanofluid. The peak value of the absorbance value of Therminol 55 is 5.42. Meanwhile, the absorbance values for CuO-Therminol 55 nanofluid with 0.1, 0.3, 0.5, 0.8, and 1.0wt%, are 5.61, 5.87, 6.01, 6.49 and 6.88 respectively. The absorbance values for Al_2_O_3_-Therminol 55 nanofluid with 0.1, 0.3, 0.5, 0.8, and 1.0 wt% are 5.60, 5.85, 6.00, 6.37, and 6.80 respectively. The absorbance value of CuO- Al_2_O_3_ (10:90) hybrid Therminol 55 nanofluid with concentrations 0.1, 0.3, 0.5, 0.8 and 1.0 wt% are 5.51, 5.63, 5.88, 6.21, and 6.79 respectively. The absorbance value of CuO- Al_2_O_3_ (20:80) hybrid Therminol 55 nanofluid with concentrations 0.1, 0.3, 0.5, 0.8 and 1.0wt% are 5.63, 5.88, 6.20, 6.52, and 6.90 respectively. The absorbance value of CuO- Al_2_O_3_ (30:70) hybrid Therminol 55 nanofluid with concentrations 0.1, 0.3, 0.5, 0.8 and 1.0 wt% are 5.67, 5.90, 6.25, 6.58, and 7.06 respectively.

The results show that, when the concentration of nanoparticles (Al_2_O_3_, CuO, or the hybrid) is raised from 0.1 to 1.0%, the absorbance also rises. This observation is consistent with the principles of the Beer-Lambert Law^54^. The Beer-Lambert Law establishes a direct correlation between the absorption of light and the characteristics of the medium through which the light is passing^55^. The rule stipulates that the absorbance A is directly related to the route length l, the concentration c of the absorbing species, and the molar absorptivity coefficient ε, as demonstrated by Eq. (6)^56^.

6 $${\text{A}}={\text{elc}}$$

Enhanced concentration can also lead to the widening of the absorption bands as a result of particle-particle interactions or an increased number of diverse absorption sites^57^. At elevated concentrations, the absorbance may not exhibit a linear relationship with concentration due to the constraints imposed by the Beer-Lambert Law, such as the presence of scattering effects or alterations in the refractive index of the solution^58^.

Recent studies have demonstrated that incorporating hybrid nanoparticles into Therminol55 significantly influences its optical properties, thereby enhancing its suitability for solar energy applications^59^. The modification of heat transfer fluids using nanotechnology has become a crucial area of research, particularly in solar thermal systems, where maximizing light absorption and minimizing thermal losses are essential for improving efficiency. The ability of hybrid nanofluids to leverage the optical advantages of different nanoparticles has led to remarkable improvements in photothermal performance, making them highly desirable for energy-intensive applications^60^.

For instance, Das et al.^61^ formulated Therminol55-based nanofluids containing MXene/Al_2_O_3_ nanocomposites and observed a significant increase in optical absorbance across the visible spectrum. This enhancement was attributed to the unique light-absorbing characteristics of MXene, known for its exceptional plasmonic resonance, and the high dispersion stability provided by Al_2_O_3_. The combination of these materials enabled a more homogeneous distribution of nanoparticles within the fluid, ensuring consistent optical performance without significant sedimentation or degradation over time. These findings align with the work of Kalidoss et al.^62^, who investigated Therminol55-TiO_2_ nanofluids and found that the addition of TiO_2_ nanoparticles led to an increase in optical density, thereby boosting the fluid’s capacity to absorb solar radiation. TiO_2_ nanoparticles are well-known for their high refractive index, which allows for superior light interaction within the fluid. This property ensures that incoming radiation is efficiently absorbed and redistributed, leading to a higher overall energy retention. Furthermore, the incorporation of hybrid nanofluids has been shown to mitigate some of the drawbacks associated with single-component nanofluids, such as limited stability, rapid sedimentation, and agglomeration^63^. By carefully optimizing nanoparticle concentration and composition, researchers have been able to achieve a balance between optical performance and fluid stability, ensuring long-term applicability in practical thermal energy systems.

Collectively, these studies underscore the immense potential of hybrid nanofluids in enhancing the optical performance of heat transfer fluids, thereby improving the efficiency of solar thermal systems. By leveraging the synergistic properties of different nanoparticle compositions, researchers continue to develop advanced heat transfer fluids that can be integrated into next-generation energy systems, further promoting the adoption of sustainable and efficient solar technologies.

### Thermal conductivity

**Fig. 10:**
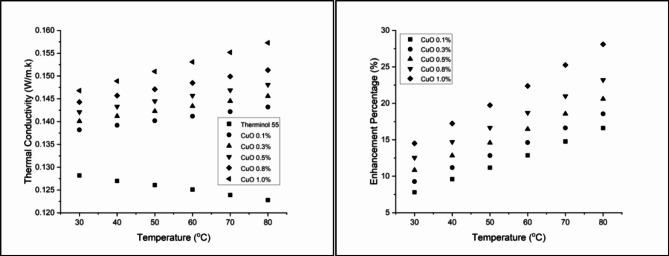
Thermal conductivity and Enhancement percentage of Copper Oxide-Therminol 55 nanofluid.

Figure 10 presents the thermal conductivity enhancement of CuO-Therminol 55 nanofluids at different concentrations and temperatures. The results indicate a steady increase in thermal conductivity with both higher nanoparticle concentration and temperature. At 0.1 wt% concentration, thermal conductivity enhancement starts at 7.80% at 30 °C and rises to 16.61% at 80 °C, highlighting the fluid’s responsiveness to temperature changes. Increasing the concentration to 0.3 wt% further improves conductivity, with enhancement values ranging from 9.28% at 30 °C to 18.57% at 80 °C, demonstrating a stronger thermal reaction^64^. Higher concentrations continue to show significant improvements, with 0.5 wt% reaching 20.60% at 80 °C, and 0.8 wt% achieving a maximum enhancement of 23.21% at 80 °C. The most substantial improvement is observed at 1.0 wt%, where conductivity rises from 14.51% at 30 °C to 28.09% at 80 °C. This enhancement is attributed to the high thermal conductivity of CuO nanoparticles and the potential formation of micro-convection currents within the fluid, further promoting heat transfer. These findings confirm that higher nanoparticle concentrations significantly improve the thermal conductivity of Therminol 55, making CuO nanofluids a promising candidate for advanced heat transfer applications^65^.

**Fig. 11:**
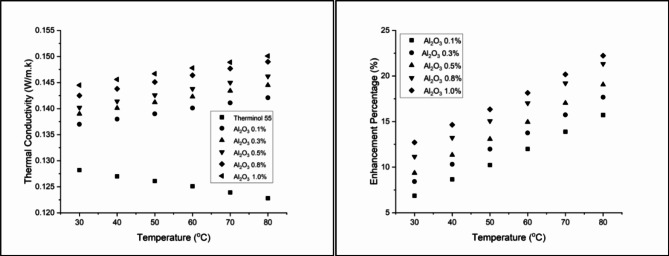
Thermal conductivity and Enhancement percentage of Aluminium Oxide-Therminol 55 nanofluid.

Figure 11 presents the thermal conductivity enhancement of Al_2_O_3_-Therminol 55 nanofluids, following a similar trend to CuO but with a lower overall enhancement due to the comparatively lower thermal conductivity of Al_2_O_3_. At 0.1 wt% concentration, thermal conductivity increases from 6.86% at 30 °C to 15.72% at 80 °C, showing a steady temperature-dependent improvement. Increasing the concentration to 0.3 wt% results in a more pronounced enhancement, reaching 17.67% at 80 °C, demonstrating the direct correlation between nanoparticle loading and heat transfer improvement^66^. At 0.5 wt%, the enhancement reaches 19.06% at 80 °C, continuing the increasing trend observed across all concentrations. The 0.8 wt% nanofluid exhibits a further rise, with thermal conductivity improving from 11.15% at 30 °C to 21.34% at 80 °C, highlighting the nanoparticles’ effectiveness at higher concentrations^67^. The maximum enhancement is observed at 1.0 wt%, where thermal conductivity improves from 12.71% at 30 °C to 22.23% at 80 °C. These results confirm that higher Al_2_O_3_ concentrations contribute to steady heat transfer improvement, though the overall enhancement remains lower than CuO-based nanofluids due to material properties^68^.

**Fig. 12:**
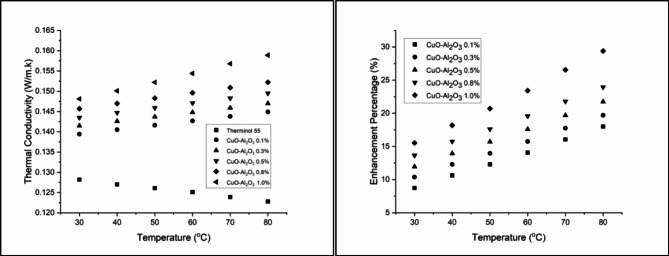
Thermal conductivity and Enhancement percentage of hybrid CuO- Al_2_O_3_ (10:90) in Therminol 55 nanofluid.

Figure 12 illustrates the thermal conductivity enhancement of CuO-Al_2_O_3_ (10:90) hybrid nanofluids across different concentrations and temperatures. At 0.1 wt%, thermal conductivity improves from 8.74% at 30 °C to 18.00% at 80 °C, showing a steady temperature-dependent increase. Increasing the concentration to 0.3 wt% leads to a more pronounced enhancement, ranging from 10.37% at 30 °C to 19.71% at 80 °C, though a slight decrease is observed at the highest temperature, suggesting an optimal range for conductivity improvement^69^. At 0.5 wt%, the enhancement continues to rise, reaching 21.74% at 80 °C, reinforcing the strong correlation between nanoparticle concentration and thermal conductivity augmentation. A further increase to 0.8 wt% results in an initial improvement of 13.65% at 30 °C, peaking at 23.94% at 80 °C, indicating that higher nanoparticle concentrations lead to greater thermal efficiency^70^. The highest improvement is observed at 1.0 wt%, where thermal conductivity increases from 15.52% at 30 °C to 29.40% at 80 °C, demonstrating the significant heat transfer enhancement achieved by hybrid nanofluids. These results confirm that CuO-Al_2_O_3_ hybrid nanofluids enhance thermal conductivity more effectively than single-component nanofluids, making them a promising alternative for advanced heat transfer applications^71^.

**Fig. 13:**
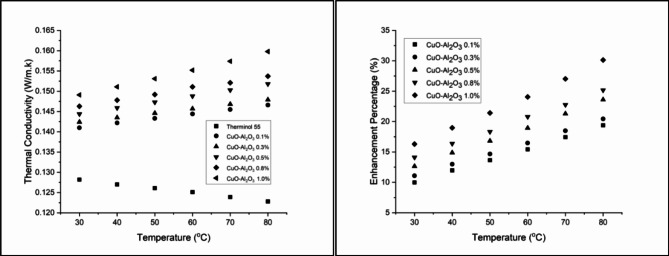
Thermal conductivity and Enhancement percentage of hybrid CuO- Al_2_O_3_ (20:80) in Therminol 55 nanofluid.

**Fig. 14:**
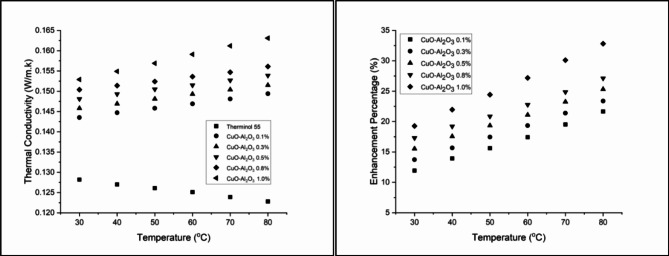
Thermal conductivity and Enhancement percentage of hybrid CuO- Al_2_O_3_ (30:70) in Therminol 55 nanofluid.

Figure 13 presents the thermal conductivity enhancement for CuO-Al_2_O_3_ (20:80) hybrid nanofluids, showing a steady increase with both concentration and temperature. At 0.1 wt%, thermal conductivity enhancement starts at 9.98% at 30 °C, reaching 19.38% at 80 °C. Higher concentrations exhibit stronger improvements, with 1.0 wt% showing the highest enhancement of 30.13% at 80 °C, confirming that higher CuO content leads to greater thermal performance^72^. Similarly, Fig. 14 illustrates the thermal conductivity trends for CuO-Al_2_O_3_ (30:70) hybrid nanofluids, following the same pattern. The enhancement at 0.1 wt% begins at 11.93% at 30 °C and rises to 21.66% at 80 °C. The highest improvement is recorded at 1.0 wt%, reaching 32.82% at 80 °C, making this the most effective hybrid formulation^73^. The results suggest that higher CuO content leads to more significant thermal conductivity improvements, aligning with the fact that CuO has a higher intrinsic thermal conductivity than Al_2_O_3_^74^.

The observed thermal conductivity enhancements can be attributed to several key mechanisms, including Brownian motion, nanoparticle clustering, the formation of nanolayers at the liquid-particle interface, and possible structural changes in the base fluid. These mechanisms enhance energy exchange efficiency by increasing the available surface area for heat transfer. Comparing these results with previous studies confirms that the addition of metallic and metal oxide nanoparticles to base fluids consistently enhances thermal conductivity, making them suitable for applications such as automobile cooling systems, electronic cooling, and solar thermal energy systems^29,75–77^. Overall, these findings demonstrate that nanoparticle type, concentration, and temperature collectively influence the extent of heat transfer enhancement, offering insights for developing optimized thermal fluids for industrial applications.

Minor deviations in temperature readings can affect the calculated thermal conductivity values, leading to slight variations in the results. To minimize this, high-precision temperature sensors with a small margin of error were employed, and multiple measurements were taken to enhance accuracy. The uncertainty in the thermal conductivity measurements was assessed based on the precision of the experimental instruments and the consistency of multiple trials. The overall uncertainty was found to be within ± 1.5%, indicating a high level of accuracy in the collected data. The uncertainty analysis table (Table 1) provides a comprehensive breakdown of potential sources of error, ensuring transparency in the reported results.

**Table 1:** The estimated uncertainties for each variable.

Parameter	Measured value	Uncertainty (%)
Temperature (℃)	30–80	± 0.2%
Thermal Conductivity (W/m·K)	0.1–0.2	± 1.5%
Sensor Accuracy	N/A	± 0.2%
Nanofluid Concentration (%)	0.1-1.0	± 0.5%

### Thermal conductivity through machine learning

The following Subsection consists of two parts. Initially, the implementation process of the proposed method under MATLAB (R2021a) software is explained. The second part discusses the regenerated results by implementing the machine learning method. Part 2 discusses more information regarding the system’s behavior based on the developed T2FNN.

#### Implementation

To implement the proposed method in the previous Section T2FNN, and MATLAB (R2021a) software are used. The whole dataset comprises 180 data based on different arrangements of temperature, concentration, and different portions of Cu: Al. The range of the temperature is between 30 and 80 °C. Also, the concentration is varied from 0 to 1%. Also, the 0:100, 10:90, 20:80, 30:70, and 100:0 portions of Cu: Al is captured. The dataset is normalized to maximize the accuracy of the proposed T2FNN during the training and testing process of the T2FNN. In the next step, the dataset is divided into 80% and 20% for training and testing purposes of the proposed T2FNN. It should be mentioned that 144 of 180 datasets are used for training.

In contrast, 36 datasets are candidates for testing purposes of the developed T2FNN. The different hyperparameters of T2FNN are examined using trial and error to reach the system’s highest efficiency. Table 2 shows the extracted hyperparameters that reached the highest performance of the developed T2FNN. It should be noted that the form of the type-2 fuzzy has a varying width of a triangular membership function with a maximum of 0.2.

**Table 2:** The hyperparameters of the proposed T2FNN.

Membership function	Epoch number	Initial step size	Step Size decrease rate	Step size increase rate	Uncertainty of the end point
3	301	7.1543e-05	0.64798	1.4931	0.2

#### Data regeneration

**Fig. 15:**
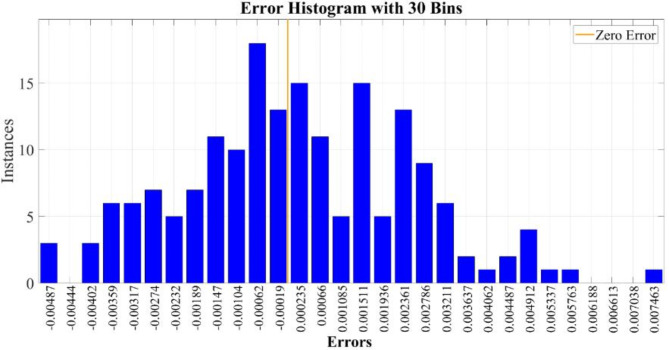
The error histogram of the proposed T2FNN during the implementation of all datasets, including training and testing.

Figure 15 depicts the distribution of errors in thermal conductivity prediction by the proposed T2FNN model, utilizing 30 bins. The error histogram reveals a left-skewed distribution, with the highest frequency at -6.2 × 10^− 4^. The mean errors for training, testing, and all datasets are 4.5954 × 10^− 5^, 6.6090 × 10^− 4^, and 1.6894 × 10^− 4^, respectively.

**Fig. 16:**
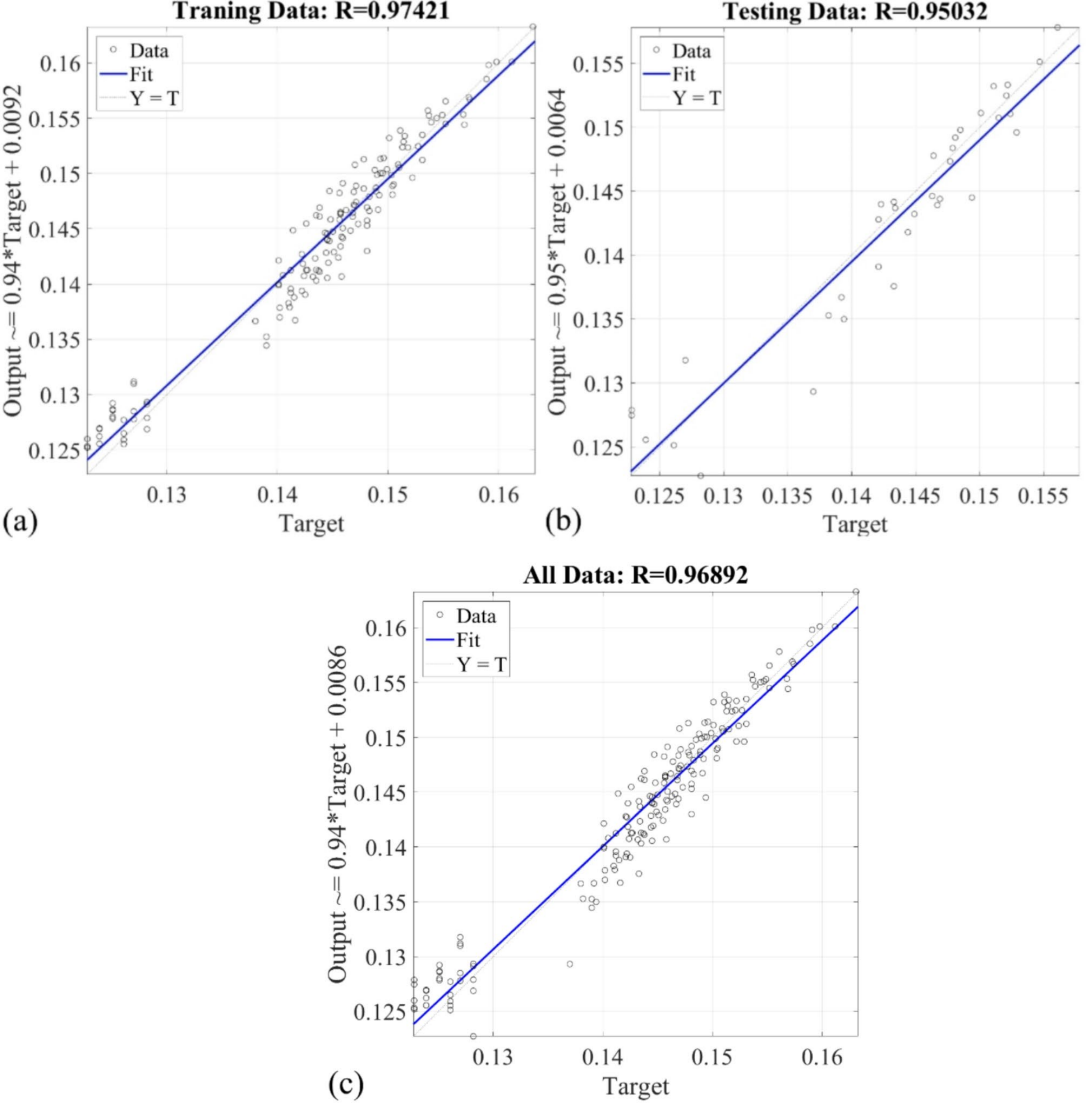
The regression of proposed T2FNN for prediction of thermal conductivity using temperature, concentration, and Cu: Al portion during (a) Training; (b) Testing; (c) All.

Figure 16a-c presents regression plots comparing experimental thermal conductivity data with T2FNN predictions for training, testing, and combined datasets. In Fig. 16a, T2FNN achieves an R-value of 0.97421 for the training dataset. Similarly, Fig. 16b displays an R-value of 0.95032 for T2FNN during testing. Figure 16c illustrates an R-value of 0.96892 for comparing experimentally obtained and T2FNN-predicted thermal conductivity across all datasets.

**Fig. 17:**
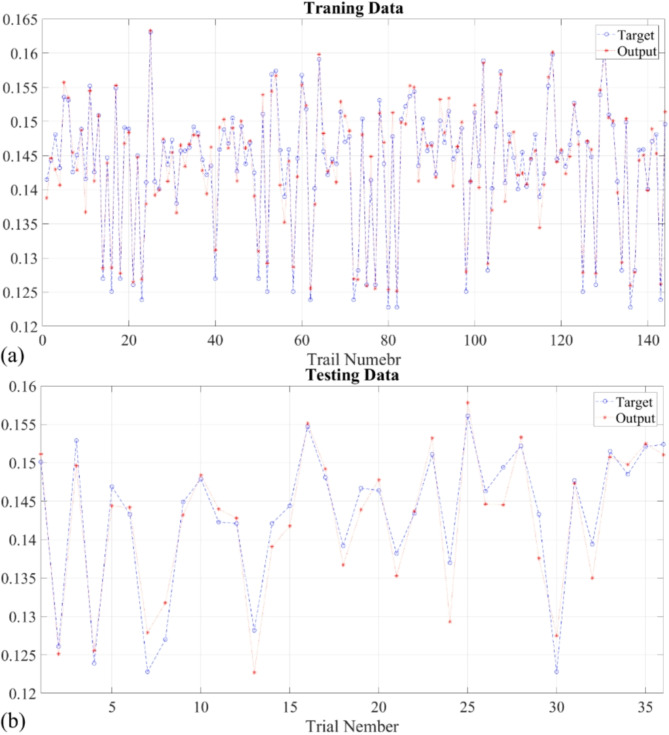
The experimental and predicted outputs of thermal conductivity during (a) training and (b) testing.

Figure 17a-b illustrates experimental (Target) and T2FNN-predicted (Output) thermal conductivity during the training and testing phases. This evaluation involves comparing experimental results with predictions from the T2FNN model. The experimental data serves as a reference to assess the accuracy of the T2FNN predictions. Figure 17a showcases the computation of thermal conductivity for 144 training samples using experimental and T2FNN-predicted values. Results in Fig. 17a reveal mean squared error (MSE) and root mean squared error (RMSE) between experimental and T2FNN-predicted thermal conductivity as 4.5059 × 10^− 6^ and 2.1 × 10^− 3^, respectively. Furthermore, Fig. 17b demonstrates similar comparisons for 36 testing samples, yielding MSE and RMSE values of 8.9444 × 10^− 6^ and 3.0 × 10^− 3^, respectively.

The integration of machine learning, specifically the T2FNN, in this study, provides a robust predictive framework for estimating thermal conductivity based on multiple input parameters. Unlike conventional empirical correlations, which rely on predefined mathematical relationships, T2FNN dynamically captures the nonlinear and complex interactions between temperature, nanoparticle concentration, and hybrid composition^78^. This adaptability enhances predictive accuracy, reducing the need for extensive experimental measurements and enabling rapid optimization of nanofluid formulations. This approach is particularly valuable for hybrid nanofluids, where the synergistic effects of different nanoparticles create intricate dependencies that traditional curve-fitting techniques struggle to model effectively^79^. By utilizing machine learning, this study presents a more generalizable and efficient method for predicting thermal conductivity, demonstrating its potential for real-world applications in industrial heat transfer systems.

Furthermore, while this study primarily focuses on the thermal conductivity and viscosity of hybrid nanofluids, real-world applications require consideration of additional parameters such as pressure drop, erosion effects, and boundary layer interactions^80^. These factors play a crucial role in the overall efficiency and feasibility of nanofluid-based thermal systems, particularly in heat exchangers, solar thermal collectors, and industrial cooling systems. Future studies can expand upon this predictive framework by integrating these additional factors, leading to a more comprehensive assessment of nanofluid performance in practical applications. By demonstrating the predictive capability of T2FNN in modeling thermal conductivity, this study lays the groundwork for a broader implementation of machine learning in nanofluid research, optimizing heat transfer performance while reducing experimental costs.

While traditional curve-fitting techniques are commonly used to model thermal conductivity, they are inherently limited by their dependence on predefined functional forms, which may not adequately capture the nonlinear and complex interactions present in hybrid nanofluids. However, T2FNN offers a more adaptable and generalizable framework for predictive analysis. Unlike empirical correlations, which require assumptions about the underlying mathematical relationships, T2FNN learns patterns directly from data, making it better suited for modeling thermal conductivity when multiple factors interact in complex and nonlinear ways. Additionally, machine learning allows for continuous improvement and expansion, meaning that additional parameters such as viscosity, pressure drop, and boundary layer effects can be incorporated in future studies without the need to re-derive entirely new correlation models.

#### Data analysis

**Fig. 18:**
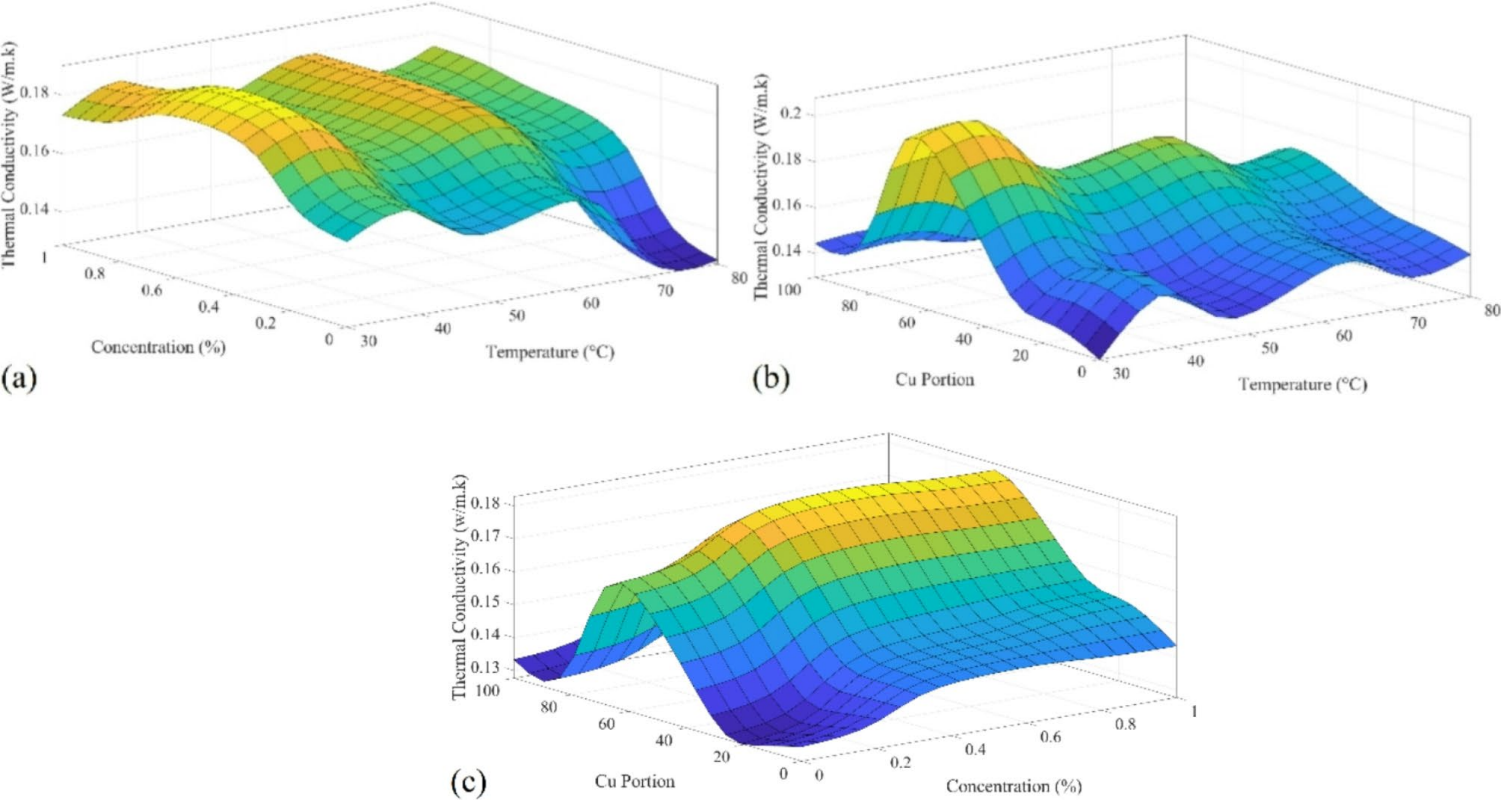
T2FNN rule surface for calculation of thermal conductivity (W/m.k) based on: (**a**) temperature (°C) and concentration (%); (**b**) temperature (°C) and Cu: Al portion; (**c**) concentration (%) and Cu: Al portion.

Figure 18a-c shows the rule surface of the extracted T2FNN in calculating the thermal conductivity based on arrangements of the input process parameters, including temperature, concentration, and Cu: Al portion. Specifically, Fig. 18a shows the variation of thermal conductivity based on the different arrangements of temperature and concentration. Based on the represented results, the highest thermal conductivity reaches lower temperatures with concentrations between 0.4% and 0.7%. However, the system’s behavior towards the temperature is not linear, and there are local optimal points at 35 and 61 °C. Figure 18b shows the variation of thermal conductivity based on the different temperatures and Cu: Al portion arrangement. Based on the represented result, pure Cu and Al reach the lowest thermal conductivity as expected during the experiment design. However, the results show that the maximum thermal conductivity reaches 60:40 of Cu: Al.

It should be noted that as the experiment design did not support the beyond 30:70 portion experiment, this point should be evaluated as a future study by conducting more experiments. However, the result for temperature is not linear, as is observed in Fig. 18a. The results of Fig. 18c support the results of previous Fig. 18a and b. The optimal range of concentrations is 0.4–0.7%. At the same time, there is a need for more investigations in the portions of Cu: Al.

### Viscosity

**Fig. 19:**
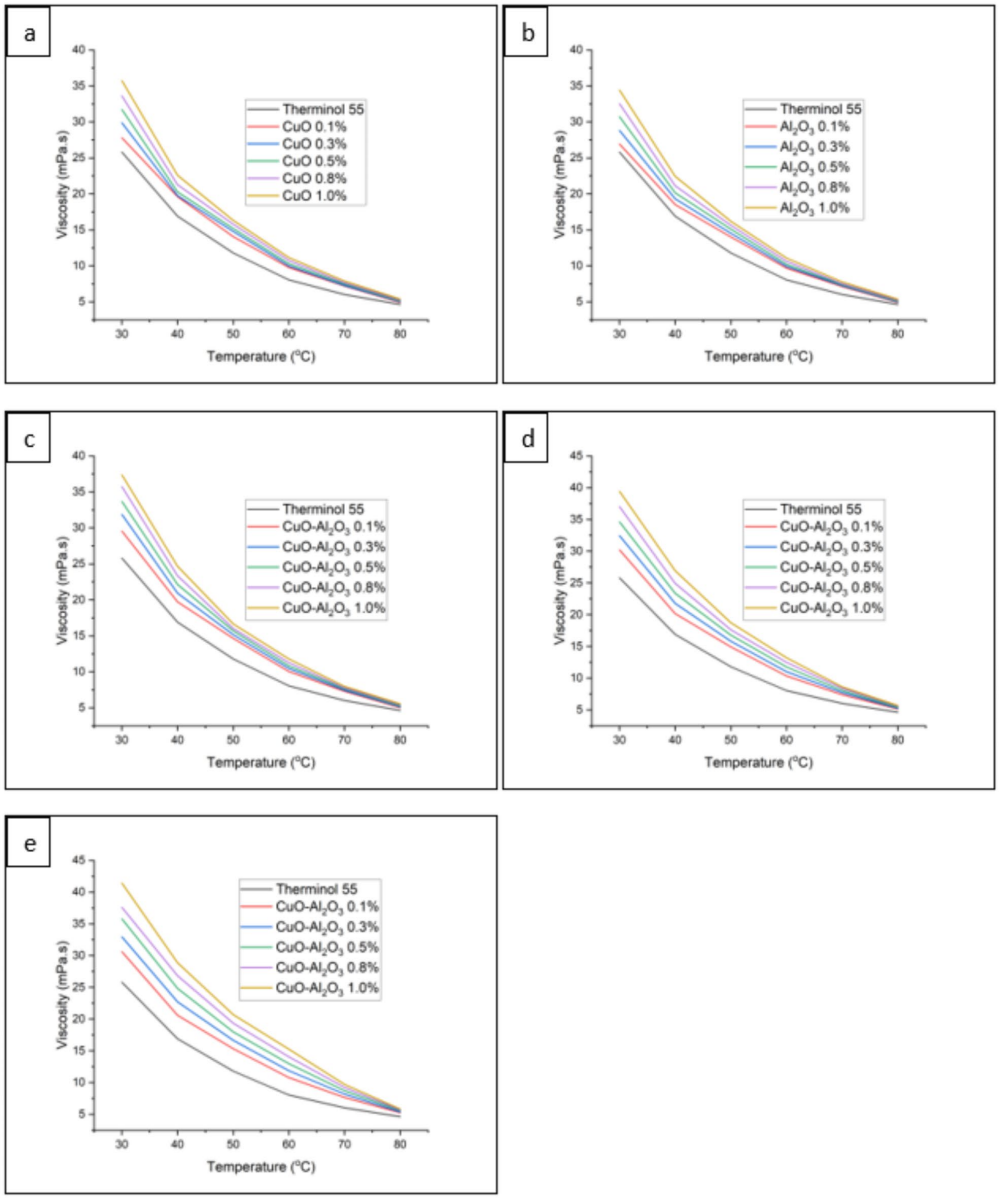
(**a**) Viscosity of CuO-Therminol 55 nanofluid with concentration 0.1wt%, 0.3wt%, 0.5wt%, 0.8wt%, and 1.0wt%. (**b**) Viscosity of Al_2_O_3_-Therminol 55 nanofluid with concentration 0.1wt%, 0.3wt%, 0.5wt%, 0.8wt% and 1.0wt%. (**c**) Viscosity of CuO- Al_2_O_3_ (10:90) hybrid Therminol 55 nanofluid with concentration 0.1wt%, 0.3wt%, 0.5wt%, 0.8wt% and 1.0wt%. (**d**) Viscosity of CuO- Al_2_O_3_ (20:80) hybrid Therminol 55 nanofluid with concentration 0.1wt%, 0.3wt%, 0.5wt%, 0.8wt% and 1.0wt%. (**e**) Viscosity of CuO- Al_2_O_3_ hybrid (30:70) Therminol 55 nanofluid with concentration 0.1wt%, 0.3wt%, 0.5wt%, 0.8wt% and 1.0wt%.

Figure 19 presents the viscosity results for CuO, Al_2_O_3_, and CuO-Al_2_O_3_ hybrid nanofluids in Therminol 55 across different concentrations and temperatures. The base fluid, Therminol 55, exhibited a decreasing viscosity trend with increasing temperature, ranging from 25.8 mPa.s at 30 °C to 4.64 mPa.s at 80 °C. A similar pattern was observed for all nanofluid formulations, where viscosity decreased as temperature increased, reducing the potential impact on flow resistance. For CuO-Therminol 55 nanofluids, the viscosity increased with nanoparticle concentration, with the highest concentration (1.0 wt%) showing the greatest increase in viscosity at all temperatures. However, the temperature-dependent reduction in viscosity remained consistent, demonstrating that higher temperatures help mitigate the increase caused by nanoparticle dispersion. A comparable trend was observed for Al_2_O_3_-Therminol 55 nanofluids, with slight variations in viscosity values depending on the nanoparticle loading.

Hybrid nanofluids, particularly CuO-Al_2_O_3_ (10:90, 20:80, and 30:70) blends, followed the same trend, with viscosity increasing with nanoparticle concentration and decreasing with temperature. The CuO-Al_2_O_3_ (30:70) hybrid nanofluid exhibited the highest viscosity values among hybrid formulations, attributed to the increased CuO content. Overall, while the addition of nanoparticles led to higher viscosity compared to the base fluid, the changes remained within reasonable limits, particularly at elevated temperatures. This suggests that hybrid nanofluids offer a balance between enhanced thermal conductivity and manageable viscosity, making them viable for practical heat transfer applications. When comparing the viscosity patterns of Therminol 55 infused with nanoparticles to the base fluid alone, it is clear that the inclusion of nanoparticles leads to an increase in viscosity at all recorded temperatures^81^. The increase in resistance to flow is a result of the improved interaction between the nanoparticles and the molecules of the fluid^82^. This phenomenon occurs at every concentration from 0.1 to 1.0%. However, as the concentration of nanoparticles rises, the increase in viscosity becomes more noticeable.

The decrease in viscosity with rising temperature, including for fluids containing nanoparticles, is an essential thermophysical characteristic of liquids^83^. Nanoparticles have an impact on the temperature-viscosity connection, but they do not change the underlying inverse relationship between these two parameters^84^. Research conducted by Awais et al.^85^ examined nanoparticle form’s impact on nanofluids’ thermal conductivity. This work provides valuable insights into thermal behavior and rheological parameters, such as viscosity. In a study conducted by Murshed et al.^86^, a thorough examination was conducted to investigate the improved thermal conductivity of TiO2 and Al2O3 nanofluids. The study also provided valuable insights into the changes in viscosity that may be compared to other findings.

Overall, the presented graphs unambiguously demonstrate the influence of adding nanoparticles and adjusting their concentration on the viscosity of Therminol 55. It is evident that the viscosity increases as the amount of nanoparticles rises. The variations in viscosity have a notable impact on applications that need accurate regulation of fluid dynamics and thermal characteristics, such as heat transfer systems^87^. The decrease in viscosity with temperature is constant for all mixes, following the accepted thermophysical behavior of fluids.

## Conclusion

This study investigated the thermal conductivity and viscosity behavior of CuO-Al_2_O_3_ hybrid nanofluids in Therminol 55 at different concentrations and weight ratios, integrating Type-2 Fuzzy Neural Network (T2FNN) modeling to predict thermal conductivity. The results demonstrated a significant improvement in thermal conductivity with increasing nanoparticle concentration and temperature. The highest enhancement was observed at 1.0 wt% CuO-Al_2_O_3_ (30:70) hybrid nanofluid, where thermal conductivity increased by 32.82% at 80 °C compared to the base fluid. Similarly, the CuO-Al_2_O_3_ (20:80) hybrid formulation at 1.0 wt% exhibited an improvement of 30.13% at 80 °C, confirming the effectiveness of hybrid nanofluids in enhancing heat transfer performance.

However, viscosity also increased with higher nanoparticle concentration, with the highest viscosity recorded at 1.0 wt% CuO-Al_2_O_3_ (30:70) reaching 41.42 mPa.s at 30 °C, gradually decreasing with temperature. Despite this, the temperature-dependent viscosity reduction mitigates excessive flow resistance, making the hybrid nanofluid practical for medium-temperature applications. Additionally, the Type-2 Fuzzy Neural Network model achieved a prediction accuracy of 96.89%, validating its effectiveness in modeling the thermal conductivity of hybrid nanofluids with minimal error.

Overall, the findings confirm that hybrid nanofluids offer superior thermal performance compared to single-component nanofluids, with CuO providing higher thermal conductivity and Al_2_O_3_ ensuring better stability. The integration of machine learning further enhances predictive capabilities, reducing experimental dependency and facilitating nanofluid optimization for heat exchangers, industrial cooling, and solar thermal systems. Future research should focus on evaluating pressure drop, erosion effects, and long-term stability to further assess the viability of hybrid nanofluids in large-scale applications.

## Data Availability

Data will be available on a reasonable request from the corresponding author.
